# Condom catheter tamponade versus surgical adjuncts for PPH in placenta previa and accreta: A prospective study

**DOI:** 10.6026/973206300222058

**Published:** 2026-04-30

**Authors:** Diksha Ambedkar, Charu Mishra, Meghna Athwani, Rina Sharma, Vijay Kumar, Yogesh K Yadav

**Affiliations:** 1 Department of Obstetrics and Gynecology, Rajarshi Dashrath Autonomous State Medical College, Ayodhya, Uttar Pradesh, India; 2 Department of Physiology, Madhav Prasad Tripathi Medical College, Siddharth Nagar, Uttar Pradesh, India; 3 Department of Community Medicine, Autonomous State Medical College, Amethi, Uttar Pradesh, India; 4 Department of Plastic Surgery, King George's Medical University, Lucknow, Uttar Pradesh, India; 5 Department of Pathology, Autonomous State Medical College, Amethi, Uttar Pradesh, India

**Keywords:** Postpartum hemorrhage (PPH), condom catheter tamponade, placenta previa, placenta accreta, uterine artery ligation

## Abstract

Postpartum hemorrhage in placenta previa and accreta is difficult to manage, especially in low-resource settings where costly devices like Bakri balloons are not readily available. Therefore, it is of interest to evaluate the effectiveness of a condom catheter in controlling hemorrhage during 29 cesarean deliveries. Patients were managed with tamponade alone or in combination with placental bed suturing and/or uterine artery ligation. Results showed significant differences in hemostasis time and blood loss. The combination of tamponade with uterine artery ligation was most effective, while placental bed suturing increased blood loss and tamponade alone resulted in delayed hemostasis. Thus, we show that condom catheter tamponade combined with uterine artery ligation is an effective and affordable option for managing hemorrhage in resource-limited settings.

## Background:

Placenta previa and placenta accreta spectrum disorders are major causes of severe postpartum hemorrhage (PPH) and contribute significantly to maternal morbidity and mortality, with a rising incidence due to increasing cesarean deliveries [1, 2-3]. These conditions result from abnormal placental implantation and adherence, leading to impaired uterine contractility and difficulty in achieving hemostasis [4]. Management of PPH in such cases is particularly challenging and often requires rapid escalation from medical therapy to mechanical or surgical interventions [5]. In cases of uncontrolled bleeding, hysterectomy may be required; however, uterus-preserving techniques are increasingly preferred [6]. These include uterine artery ligation, compression sutures and balloon tamponade [7, 8]. Condom catheter tamponade has emerged as a simple, cost-effective and minimally invasive method, especially useful in low-resource settings, with encouraging success rates reported in clinical studies [9]. Although balloon tamponade is well established for uterine atony, in placenta previa and accreta, tamponade alone may be insufficient and may require additional surgical adjuncts such as compression sutures, arterial ligation, or hysterectomy [10].

Recent evidence emphasizes individualized and stepwise management approaches to achieve effective hemorrhage control while preserving the uterus [11]. However, the use of condom catheter tamponade in placenta previa and accreta is sparsely reported. Therefore, prospective evaluation comparing tamponade alone versus its combination with surgical adjuncts is necessary to optimize management strategies in these high-risk cases [12]. Therefore, it is of interest to study of condom catheter tamponade alone versus surgical adjuncts for PPH in placenta previa and accrete.

## Materials and Methods:

This prospective interventional study was conducted at Rajarshi Dashrath Autonomous State Medical College, Ayodhya, from February 2022 to January 2025.The objective was to compare outcomes with respect to hemostasis time and blood loss using condom catheter tamponade alone versus its use in combination with placental bed suturing and/or uterine artery ligation in the management of postpartum hemorrhage due to placenta previa and placenta accreta. The study included 29 patients undergoing lower segment caesarean section (LSCS) with placenta previa or accreta diagnosed either pre-operatively or intra-operatively. These patients were allocated into four groups: tamponade alone (T), tamponade with placental bed suturing (T+PS) and tamponade with uterine artery ligation (T + UAL) and a combination of all three (T+UAL+PS).Patients who delivered vaginally, cases of atonic and traumatic PPH and cases of placenta percreta were excluded from the study. Serving a predominantly rural population, condom catheter balloon tamponade was used as a cost-effective alternative to commercial balloon devices. Blood loss was estimated by the volumetric method. The Shapiro-Wilk test was applied to assess the normality of the variables, which were found to be non-normally distributed. Consequently, the non-parametric Kruskal-Wallis test was used for further analysis.

## Results:

Kruskal-Wallis test showed that the T+UAL group had the lowest mean rank for hemostasis time (12.55) and relatively lower blood loss (12.2), indicating the fastest bleeding control. In contrast, groups involving placental bed suturing showed higher mean ranks, reflecting longer hemostasis times and greater blood loss. Tamponade alone was associated with minimal blood loss (8.3) but delayed hemostasis (15.1). Table 1 (see PDF) presents a comparative analysis of different procedural combinations used for controlling postpartum hemorrhage, evaluated using the Kruskal-Wallis test. A statistically significant difference was observed among the groups in both the time required to achieve hemostasis and the estimated blood loss (p < 0.05). The tamponade combined with uterine artery ligation (T+UAL) group demonstrated the lowest mean rank for time to arrest bleeding (12.55), indicating the most rapid hemostatic control among all groups. This group also show relatively lower estimated blood loss (mean rank 12.2), suggesting effective vascular control through arterial ligation. In contrast, procedural combinations involving placental bed suturing (PS), either alone or in combination with uterine artery ligation, exhibited higher mean ranks for both hemostasis time and blood loss. This reflects prolonged operative duration and increased intraoperative bleeding, likely due to extensive placental bed manipulation and suturing. Tamponade alone (T) was associated with the lowest mean rank for estimated blood loss (8.3), indicating minimal bleeding; however, it demonstrated a higher mean rank for hemostasis time (15.1), suggesting delayed bleeding control when used as a sole intervention. Figure 1 (see PDF) graphically illustrates the comparative effectiveness of the different procedural groups in terms of hemostasis time and estimated blood loss. The T+UAL group consistently demonstrated the shortest time to achieve hemostasis along with relatively lower blood loss, highlighting the advantage of combining mechanical tamponade with vascular control. Groups involving placental bed suturing (T+PS and T+UAL+PS) showed longer operative times and higher blood loss, reflecting increased surgical complexity and tissue handling. The tamponade-only group experienced drastic decrease in blood loss due to immediate insertion after uterine incision closure, whereas in other groups it was applied only after uterine artery ligation or placental bed suturing; however, achieving complete hemostasis with tamponade alone took longer without affecting the patient's hemodynamic status. The findings support a stepwise approach to postpartum hemorrhage management, wherein mechanical tamponade provides initial bleeding control, while uterine artery ligation enhances hemostatic efficiency by reducing uterine perfusion. The addition of placental bed suturing, although useful in selected cases, may prolong surgery and increase blood loss.

## Discussion:

While intrauterine balloon tamponade is well studied for atonic PPH, evidence regarding its use in placenta previa and accreta is limited, with even fewer studies focusing on condom catheter use. This study aimed to address this gap by evaluating the effectiveness of condom catheter tamponade, both alone and in combination with other techniques, in managing hemorrhage associated with placenta previa and accreta. This study demonstrated that tamponade, either alone or combined with uterine artery ligation, achieved hemostasis more quickly and with reduced blood loss, indicating superior effectiveness in managing postpartum hemorrhage (PPH) associated with placenta previa or accreta. As shown in Figure 1 (see PDF), although tamponade alone took longer to achieve complete hemostasis, it led to an immediate reduction in bleeding without compromising the patient's hemodynamic stability. In contrast, procedures involving placental bed suturing (PS), such as T+PS or T+PS+UL, were associated with increased operative time and higher blood loss due to tissue cut-through and the need for multiple attempts to achieve hemostasis. Uygur*et al.*[13], using the BT-Cath®balloon, reported an 85% success rate in cases of placenta previa. The use of the Bakri balloon tamponade resulted in a success rate of 88% in placenta previa in the study by Pingray*et al.*[14] and 82.9% in placenta accreta in the study by Hu*et al.*[15]. Joshi*et al.* [16] noted a lower success rate (72.8%) of intrauterine balloon tamponade (IUBT), with coagulopathy and placenta accreta identified as adverse prognostic factors. Kellie*et al.*[17] found that combining uterine artery ligation, Bakri balloon and B-Lynch suture offered the highest success in achieving hemostasis. Logistic regression by Hu*et al.*[15] also showed that pre-balloon interventions- such as uterine artery embolization (UAE), artery ligation and compression sutures-significantly increased the effectiveness of tamponade (OR = 3.9; p = 0.002). Complications such as secondary PPH, infection, sepsis, cervical trauma, leakage, or expulsion were not reported in our study. The findings of the present prospective study support the growing body of evidence advocating the use of uterine balloon tamponade as an effective first-line mechanical intervention for controlling postpartum hemorrhage (PPH), particularly when medical management fails [18]. Systematic reviews and evidence-based guidelines have consistently emphasized early implementation of tamponade techniques to reduce the need for more invasive surgical procedures. Recent high-quality clinical trials and large cohort studies have demonstrated that timely mechanical tamponade can significantly decrease blood loss and improve maternal outcomes in severe PPH, including cases complicated by abnormal placentation [19, 20]. However, the effectiveness of tamponade alone appears to be variable in placenta previa and placenta accreta spectrum disorders due to extensive placental bed vascularity and impaired myometrial contraction. Several studies have shown that adjunctive surgical measures, such as compression sutures, stepwise devascularization, or arterial ligation, may be required when tamponade alone fails to achieve sustained hemostasis [21, 22]. These findings are consistent with the present study, which observed improved hemorrhage control when condom catheter tamponade was combined with surgical adjuncts in selected high-risk cases. Evidence from Scandinavian and European studies has shown that conservative surgical approaches, when applied judiciously, can preserve uterine integrity while effectively managing hemorrhage, thereby reducing the rate of peripartum hysterectomy [23]. This aligns with contemporary obstetric practice, which prioritizes fertility preservation whenever clinically feasible. Earlier observational studies have also demonstrated that delayed escalation to surgical management may increase the risk of massive transfusion and maternal morbidity [24]. Therefore, a stepwise protocol that integrates early tamponade with timely surgical intervention appears to offer the most balanced approach for managing PPH in placenta previa and accreta. Furthermore, recent prospective and diagnostic studies have emphasized the importance of individualized decision-making informed by intraoperative findings, hemodynamic stability and the response to initial tamponade [25]. Such tailored management strategies are crucial in optimizing outcomes while minimizing unnecessary surgical morbidity. Overall, the available evidence supports a combined approach wherein condom catheter tamponade serves as an effective initial measure, with surgical adjuncts employed promptly when required, particularly in cases of placenta previa and accreta associated with severe PPH [26]. Limitations include a small sample size, which may limit generalizability; a single-center, non-randomized design introduces potential bias; Long-term outcomes were not assessed. Early and effective control of postpartum hemorrhage in placenta previa and accreta is critical to reduce maternal morbidity and mortality. The findings highlight the role of condom catheter tamponade as a valuable first-line mechanical intervention, with improved outcomes when combined with timely surgical adjuncts in high-risk cases. Adoption of a stepwise, evidence-based approach can enhance uterine preservation while ensuring adequate hemorrhage control. Condom catheter tamponade should be considered early in the management of postpartum hemorrhage associated with placenta previa and accreta, particularly when medical therapy is insufficient. Clinicians should be prepared to promptly escalate to adjunctive surgical measures such as devascularization when tamponade alone fails. Implementation of standardized protocols and multidisciplinary decision-making is essential to optimize maternal outcomes and minimize the need for peripartum hysterectomy.

## Conclusion:

Condom catheter tamponade is an effective uterus-preserving method for controlling PPH in placenta previa and accreta. Adding uterine artery ligation accelerates hemostasis and reduces blood loss, while tamponade alone lowers immediate bleeding without adversely impacting hemodynamic stability. Moreover, placental bed suturing adds complexity and blood loss without clear benefit.

## Advancement of Knowledge:

The study demonstrates that condom catheter is an effective, low-cost alternative to expensive devices in placenta previa/accreta management. Tamponade combined with uterine artery ligation yields optimal results; however, tamponade alone also significantly reduces blood loss without compromising hemodynamic stability.
